# Cellular Basis of Embryonic Hematopoiesis and Its Implications in Prenatal Erythropoiesis

**DOI:** 10.3390/ijms21249346

**Published:** 2020-12-08

**Authors:** Toshiyuki Yamane

**Affiliations:** Department of Stem Cell and Developmental Biology, Mie University Graduate School of Medicine, 2-174 Edobashi, Tsu 514-8507, Japan; yamanet@doc.medic.mie-u.ac.jp

**Keywords:** erythropoiesis, primitive erythrocyte, embryonic hematopoiesis, fetal hematopoiesis, yolk sac, fetal liver, hematopoietic stem cell, transcription factor, developmental switch

## Abstract

Primitive erythrocytes are the first hematopoietic cells observed during ontogeny and are produced specifically in the yolk sac. Primitive erythrocytes express distinct hemoglobins compared with adult erythrocytes and circulate in the blood in the nucleated form. Hematopoietic stem cells produce adult-type (so-called definitive) erythrocytes. However, hematopoietic stem cells do not appear until the late embryonic/early fetal stage. Recent studies have shown that diverse types of hematopoietic progenitors are present in the yolk sac as well as primitive erythroblasts. Multipotent hematopoietic progenitors that arose in the yolk sac before hematopoietic stem cells emerged likely fill the gap between primitive erythropoiesis and hematopoietic stem-cell-originated definitive erythropoiesis and hematopoiesis. In this review, we discuss the cellular origin of primitive erythropoiesis in the yolk sac and definitive hematopoiesis in the fetal liver. We also describe mechanisms for developmental switches that occur during embryonic and fetal erythropoiesis and hematopoiesis, particularly focusing on recent studies performed in mice.

## 1. Introduction

Erythrocytes are observed in jawed vertebrates and jawless fishes (lampreys and hagfish) [1]. Because passive diffusion of oxygen into plasma cannot fulfill the oxygen demands of the complicated tissues of large animals, the invention of erythrocytes, which are specialized to produce hemoglobins for the transport of oxygen, has been an innovative event during vertebrate evolution [2]. Hemoglobin is a tetramer consisting of α-chain and β-chain subunits, which might evolve from monomer globin with simple genetic modifications [3]. Gene duplication and genome duplication play a pivotal role in the diversification of hemoglobin genes [4]. In reptiles, birds, and mammals, α globin genes and β globin genes are located on distinct chromosomes. Intriguingly, globin genes in each chromosome are aligned tandemly by the order of expression along with development in mammals [4].

In humans, α-globin genes are arranged in the following order: ζ, α_2_, and α_1_. Similarly, α-globin genes are arranged as ζ, α_1_, and α_2_ in mice. For the β-locus, globin genes are arranged in the order of ε, ^G^γ, ^A^γ, δ, and β in humans and ε_y_, β_H1_, β_major_, and β_minor_ in mice. In humans, ζ and ε are the first globin genes to be expressed only during early embryogenesis. Along with fetal development, these genes are replaced by α and γ genes. Although α genes continue to be expressed throughout the lifetime, γ genes are replaced by the β gene after birth. The δ gene is also expressed after birth; however, δ globin constitutes only a minor hemoglobin because only a small amount of δ globin is produced from the gene [5]. Hence, globin-switching occurs twice in humans. In contrast, mice undergo globin-switching only once. In early embryonic life, mouse erythrocytes express ζ, ε_y_, and β_H1_ globins, but only low amounts of α, β_major_, and β_minor_ globins. In the mid-gestational stage, ζ, ε_y_, and β_H1_ globin genes are silenced and replaced by α, β_major_, and β_minor_ globins [6,7]. These late-fetal/adult globin genes continue to be expressed throughout life. It is known that globin molecules expressed only during embryonic and fetal life have higher affinity for oxygen compared to adult globins, which presumably allows for efficient maternal–embryonic gas exchange in the placental environment [8].

In this decade, pathbreaking studies have uncovered molecules involved in globin-switching. In particular, it was revealed that transcription factors BCL11A and ZBTB7A (also known as LRF) play pivotal roles in repressing embryonic/fetal globin expression in both humans and mice [9,10,11,12]. In addition, a recent study reported that downregulation of ATF4, a transcription factor involved in cellular stress response, may take part in the re-expression of human γ-globins in the β-thalassemia state [13]. Mechanisms linking transcription factors to epigenetic control of the globin locus have been well documented [5,14].

Besides the well-recognized role of erythrocytes in the transport and exchange of gas through hemoglobins, it is not widely recognized that erythrocytes have regulatory immune functions. It is known that primate erythrocytes express complement receptor 1 (CR1/CD35) on its surface. CR1 on primate erythrocytes seems to be crucial for the transport of immune complexes to the liver and spleen, where tissue macrophages ingest and destroy the complex, thereby preventing immune complex diseases such as systemic lupus erythematosus [15]. In addition, it is reported that neonatal erythrocytes exert immunoregulatory function through arginase-2, which may contribute to the suppression of immune response to infectious agents and fetomaternal tolerance [16,17,18].

Hematopoietic stem cells continue to self-renew and differentiate into mature blood cell lineages throughout their lifetime [19]. Recent lineage tracing technologies have reconfirmed the multipotent nature of hematopoietic stem cells [20,21]. Except for some tissue macrophages, B-1 B cells, and epidermal γδ T cells, maintenance of peripheral pools of mature blood cell lineages is dependent on supplies from hematopoietic stem cells [7]. Besides the dependency of erythrocyte production on hematopoietic stem cells in adult animals, hematopoietic stem cells with long-term repopulating ability do not appear until the late embryonic/early fetal stage during development [7]. Until a sufficient number of hematopoietic stem cells are produced in the fetal liver to supply adult-type (definitive) erythrocytes, embryo/fetus-specific erythrocytes, called primitive erythrocytes, which are produced in the extra-embryonic yolk sac tissue, play a pivotal role in transporting oxygen to embryonic and fetal tissues. In fact, mouse embryos lacking primitive erythrocytes do not survive beyond the late embryonic/early fetal stage. Hence, primitive erythrocyte-mediated gas transport and exchange is critical for embryonic and fetal development [7]. In addition, recent studies indicate that multipotent hematopoietic progenitors derived from the yolk sac contribute transiently to definitive erythropoiesis in the fetal liver. The processes of yolk sac primitive and fetal liver definitive erythropoiesis have been studied in detail in mice. In this review, we discuss how primitive and definitive erythrocytes are produced during mouse embryonic and fetal life, especially focusing on their developmental origins. In addition, we briefly describe human embryonic and fetal erythropoiesis at the end of this review.

## 2. Features of Primitive Erythropoiesis

Primitive erythrocytes are the first hematopoietic cells observed during vertebrate ontogeny. Primitive erythropoiesis constitutes the earliest and transient erythropoiesis during development. Primitive erythrocytes express the embryonic and fetal globins mentioned above, circulate in embryonic and fetal blood, disappear around parturition, and are eventually replaced by hematopoietic stem-cell-derived erythrocytes [6].

Primitive erythropoiesis predominantly occurs in the yolk sac in mammalian embryos. The yolk sac is an extra-embryonic membranous tissue consisting of two layers: a hypoblast-derived visceral endoderm layer and an epiblast-derived extra-embryonic mesoderm layer. Some animals with multiple conceptuses, including mouse, rat, rabbit, and guinea pig, form “egg cylinders” within the limited space of uterus. Each egg cylinder contains a U-shaped embryo with the neuroectoderm inside and the gut endoderm outside [7,22]. In this type of animal, a turning (axial rotation) eventually reverses the positioning of the embryo into a standard topology. By this movement, embryos are completely enveloped by the yolk sac. In most animals, including humans, the yolk sac does not envelope embryos, but this membranous tissue is attached to the embryo [7,22]. Primitive erythroid cells were initially observed as cell aggregates in the yolk sac. These eythroid cell aggregates are called blood islands until vasculatures are firmly established within the yolk sac [6,7]. Phylogenetically, primitive erythropoiesis in the yolk sac is observed as early as in some ray-finned fishes [7].

Primitive erythrocytes are large in size and enter into circulation in their nucleated form. This is in striking contrast to adult erythrocytes of mammals, which are smaller in size and circulate in its enucleated form. Primitive erythropoiesis is most well-described in mice compared to other animals. Cellular makers for primitive erythrocytes are observed as early as embryonic day 7.5 (E7.5) at the presomite-headfold stage of mouse development in the cell aggregates of blood islands [6,23]. The primitive erythrocytes enter into circulation through the vitelline vessel when the heartbeat starts [24]. Although primitive erythrocytes circulate in the embryonic/fetal circulation in a nucleated form initially, it is reported that these cells finally undergo enucleation during E12.5 to E16.5 in mice [25]. Macrophages in the fetal liver may promote enucleation of these erythrocytes because it has been reported that mice lacking DNase II became paler as early as at E14.5 and died in utero due to severe anemia because of erythrocyte-extrinsic reasons [26].

Erythropoietin receptor and KIT signaling are essential for erythropoiesis in adult mice. However, signaling from these cytokine receptors seems dispensable for primitive erythropoiesis, although these receptors are expressed in cells in the yolk sac [27,28,29,30]. In contrast, FLK1 (also known as VEGFR2) is required for primitive erythropoiesis [31]. Hence, cytokine dependency is different between primitive and adult erythropoiesis.

## 3. Complicated Nature of Late Embryonic/Early Fetal Hematopoiesis

It has long been thought that hematopoiesis occurs in two phases during development: transient primitive hematopoiesis (primitive erythropoiesis plus some macrophage lineage production) and “definitive” hematopoiesis, which produces all lympho-myeloid lineages and adult-type erythrocytes. However, the more complicated nature of embryonic/fetal hematopoiesis has been revealed in the past two decades. Many of these findings were observed in studies using mouse models, including that there are intermediate cell populations between primitive hematopoiesis and hematopoietic stem-cell-originated definitive hematopoiesis.

As mentioned above, primitive erythropoiesis starts as early as E7.5 in the blood islands of the yolk sac in mice. In contrast, long-term repopulating hematopoietic stem cells were first observed at E10.5–E11.5 in multiple locations in mice, including the papa-aortic region, placenta, vitelline and umbilical arteries, head region, and yolk sac [7]. However, the number of hematopoietic stem cells in one embryo is estimated to be less than one at this stage [32]. Extensive proliferation of hematopoietic stem cells occurs after these cells move into the fetal liver. Some types of transient multipotent hematopoietic progenitors are found in the yolk sac. These transient multipotent progenitors probably fill the gap until a sufficient number of hematopoietic stem cells are produced to supply whole blood cells.

**Figure 1:**
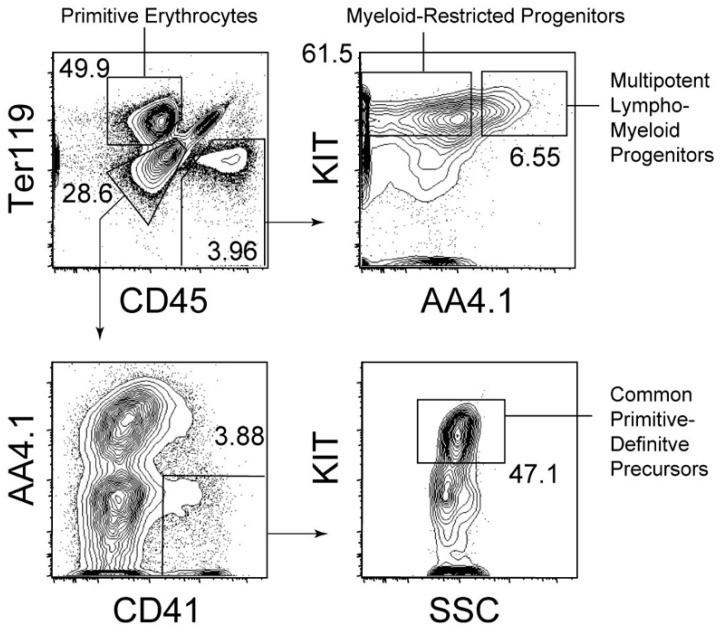
Hematopoietic cell fractions in the E9.5 yolk sac. Representative cell gating strategies to identify hematopoietic cell populations in the E9.5 yolk sac are shown. Numbers in plots indicate percentages of the cells in the gate within the parental cell fraction.

Hematopoietic progenitors with lympho-myeloid potency, but lacking self-renewal capability, are present before hematopoietic stem cells appear in the body, especially in the yolk sac of E9.5 mouse embryos [33,34]. Hence, the emergence of multipotency in the hematopoietic system precedes that of self-renewal capability. A recent study suggested that EZH1, an epigenetic regulator, is involved in halting hematopoietic stem cell activity in the yolk sac and embryos [35]. In parallel with lympho-myeloid progenitors, myeloid-restricted progenitors are abundantly present in the E9.5 yolk sac [33]. Cell surface marker expression and cell identification strategies used for the isolation of these progenitors are described in Figure 1. As shown in Figure 1, the expression of AA4.1 within the CD45^+^ cell population distinguishes lympho-myeloid progenitors from myeloid-restricted progenitors. Yolk sac hematopoietic progenitors, except for primitive erythroblasts, are often referred to as erythroid-myeloid progenitors [36]. AA4.1^-^ myeloid-restricted progenitors are the closest to so-called erythroid-myeloid progenitors because these cells lack the ability to generate primitive erythrocytes, but can generate granulocytes, monocytes/macrophages, and adult-type (definitive) erythrocytes, that is, cells solely expressing adult-type hemoglobins [33]. In addition to these progenitors, lympho-myeloid progenitors lacking the capability of erythroid and megakaryocytic differentiation have been reported to be present in the early fetal liver [37]. A small number of this type of progenitors may also be present in the E9.5 yolk sac [37].

Intriguingly, multipotent lympho-myeloid progenitors in the E9.5 yolk sac preferentially generate B-1 B lymphocytes, although these cells still possess little potency to generate follicular B and marginal zone B lymphocytes [38]. B-1 B lymphocytes are known to produce IgM antibodies for polysaccharides and phospholipids. B-1 B cells have apparently different specificity from follicular B cells that produce antibodies mainly against protein antigens and undergo class-switching to other antibody subclasses. First-wave antibody production may be specialized to non-follicular B antibody repertoire because the follicular B antibody repertoire can be obtained from the mother in the form of IgG subclasses through the placenta [7]. In addition, a recent study reports that epidermal γδ T cells may also originate from the yolk sac [39]. Epidermal γδ T cells are known to predominantly express T cell receptor with a Vγ5Vδ1 allele. These T cells are involved in immunological barriers and wound healing of the skin through the secretion of cytokines [40,41]. It is noteworthy that epidermal γδ T cells are solely derived from embryonic stage and self-renew in the skin independently of hematopoietic stem cells [39].

## 4. Cellular Origin of Primitive Erythrocytes in Mice

As mentioned above, primitive erythrocytes were thought to be separate cell types from other lympho-myeloid hematopoietic cell lineages. Some studies have suggested bipotential hematopoietic activity for primitive erythrocytes and multilineage hematopoiesis. Although these studies clearly showed that single cells could generate both primitive erythrocytes and multiple myeloid lineages, including adult-type erythrocytes, the developmental stages of cells showing bipotential capability were unclear [42]. However, recent studies have shown that a committed hematopoietic cell population serves as a common precursor for primitive erythrocytes and lympho-myeloid lineages. These earliest hematopoietic progenitors are found in the CD41^+^CD45^−^ cell stage and lack endothelial potency [29]. Hence, these progenitors are committed hematopoietic cells. At the single-cell level, these cells can generate primitive erythrocytes and B progenitors that undergo gene rearrangements; therefore, these cells serve as common primitive-definitive precursors [29]. However, these cells still lack self-renewal capability [29]. These common precursors for primitive erythrocytes and lympho-myeloid lineages are abundantly present through the E8 to E9 yolk sac. Therefore, these cells seem to be a major source of primitive erythrocytes in the E8 to E9 yolk sac. However, the exact in vivo potency of these progenitors is not deciphered here as this study is based on ex vivo detection of globin mRNA and gene rearrangement [29,43]. However, further studies using in vivo tracking technologies without disrupting embryos may answer this question. Detailed cell surface marker expression and cell identification strategies used for the isolation of these progenitors are summarized in Figure 1.

**Figure 2:**
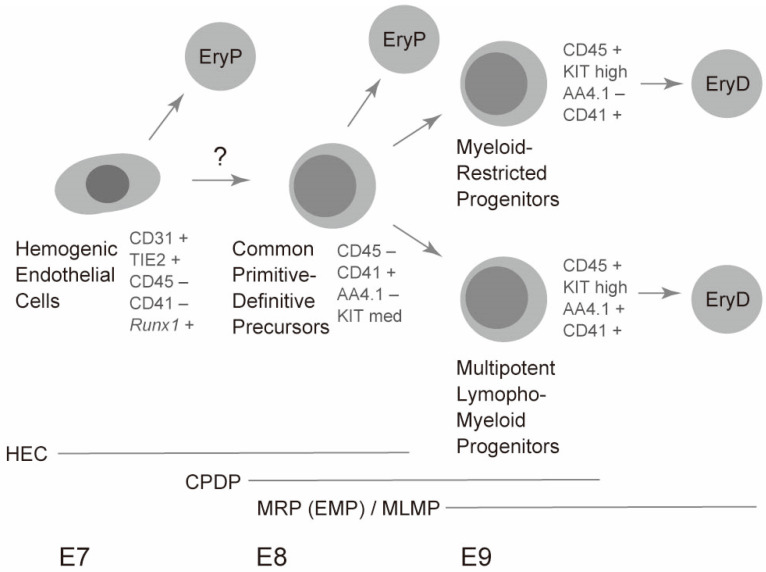
A model of erythropoiesis in the yolk sac. Timeline of erythropoiesis is schematically described along with the embryonic day of mouse development (E7–E9). Representative marker expression is shown along with the cell population names. Periods when each cell population are present are shown by horizontal bars. EryP, primitive erythrocytes; EryD, definitive (adult-type) erythrocytes; HEC, hemogenic endothelial cells; CPDP; common primitive-definitive precursors; MRP, myeloid-restricted progenitors; EMP, erythroid-myeloid progenitors; MLMP, multipotent lympho-myeloid progenitors.

Interestingly, the common precursors for primitive erythrocytes and lympho-myeloid cells express modest levels of erythroid transcription factors. In particular, the expression of *Gata1*, *Scl/talI*, and *Klf1*, which form an erythroid core transcriptional network by co-occupying erythroid-specific genes, was approximately 1/2, 1/2, and 1/10 of CD71^+^Ter119^−^ primitive erythroblasts in the yolk sac common precursors, respectively [30,44]. The expression of these transcription factors likely enables the immediate generation of primitive erythrocytes in one day from the common precursors. Simultaneously, the yolk sac common precursors express essential transcription factors for definitive hematopoiesis, including *Gata2*, *Meis1*, *Myb*, *Pu.1*, and *Runx1* [30]. Expression of these definitive hematopoiesis genes seems to ensure differentiation into lympho-myeloid lineages. Hence, the balance between primitive erythroid transcription and lympho-myeloid transcription factors likely affects the alternative fate of the common precursors. Intriguingly, lympho-myeloid progenitors in the E9 yolk sac express much lower levels of the above-mentioned core erythroid transcription factors, whereas lympho-myeloid progenitors express approximately 2-fold more *Myb* and *Pu.1* genes compared to the common precursors. In particular, *Pu.1* seems critical for the differentiation of lympho-myeloid progenitors. This transcription factor promotes the process by repressing *Gata1*, *Scl/talI*, and *Klf1* genes, thereby blocking the primitive erythroid program [30]. Mutual antagonism of GATA1 and Pu.1 are well documented [44]. Therefore, the balance between *Gata1* gene and *Pu.1* gene may influence the alternative fate of common precursors. It is noteworthy that adult type-erythrocytes are generated solely from multipotent lympho-myeloid progenitors and myeloid-restricted progenitors, but not directly from the common precursors. Therefore, the common precursors alone can generate two distinct waves of primitive erythropoiesis and definitive erythropoiesis (Figure 2) [29].

In the sections above, “committed” hematopoietic progenitors have been discussed in isolation. Hematopoietic cells are thought to arise from endothelial cells [45]. The term “hemogenic endothelial cells” is used to describe endothelial cells with hematopoietic capability. However, the term has often been misused by researchers. Even committed hematopoietic progenitors are referred to as hemogenic endothelium in some cases. In contrary to these confusing uses of the term, hemogenic endothelial cells are clearly defined in a report as the cells that express CD31 (an endothelial marker) and *Runx1* gene (hematopoietic transcription factor), but lack CD41 and CD45 (hematopoietic cell markers) expression [46]. The authors described that the cells have the potential to form endothelial tubes in vitro. Therefore, these cells seem to be present in the uncommitted state.

As mentioned above, primitive erythroid cells are found as early as E7.5 during mouse development. Clear expression of β_H1_ has already been reported at this stage [6,23]. This observation raises a question about the origin of precursors of the E7.5 primitive erythroid lineage, because CD41 expression seems to be initiated at a similar timing as globin expression [23]. Hemogenic endothelium may explain the very early presence of primitive erythroid cells. Cells positive for TIE2 (an endothelial marker), but negative for CD41, are reported to produce β_H1_-expressing primitive erythroid cells as well as endothelial structure [23]. It is still unknown whether hemogenic endothelial cells go through the common precursor stage described above, and then differentiate into primitive erythroid cells. Further studies are necessary to answer this question. A current model for the generation of primitive erythrocytes is shown in Figure 2. Moreover, a recent study reported the presence of yolk sac hemogenic endothelial cells specialized in generating solely the definitive lineages, but not the primitive erythrocytes, suggesting an early segregation of primitive erythrocytes and definitive lineages [47]. However, further studies need to be conducted to precisely determine how different types of progenitors contribute to primitive erythropoiesis and definitive lymphohematopoiesis in the early yolk sac along with embryonic stages over time [7,48].

## 5. Erythropoiesis and Hematopoiesis in the Fetal Liver

The fetal liver is the largest site of fetal erythropoiesis and hematopoiesis. The major cell population that initially colonizes the fetal liver at E10–E11 is yolk-sac-originated cells. This is because the vitelline vein from the yolk sac directly drains into the fetal liver and this movement occurs when other hematopoiesis sites such as the aortic region and placenta do not contain a large pool of hematopoietic cells [7]. Therefore, myeloid-restricted progenitors (erythroid-myeloid progenitors) and multipotent lympho-myeloid progenitors from the yolk sac are likely to be initial sources of fetal liver transient definitive (late fetal/adult-type) erythropoiesis and hematopoiesis. This is also supported by the observation that most of the blood cell lineages except primitive erythrocytes have a CD45^+^KIT^high^ phenotype at this stage but lack a Sca1 hematopoietic stem cell marker. In addition, up to 10% of CD45^+^KIT^high^ cells express AA4.1, a lympho-myeloid progenitor marker, at this stage (Figure 2) [7].

After the first wave of erythropoiesis and hematopoiesis, hematopoietic stem cells generated at multiple sites, including the aorta, placenta, and yolk sac, migrate into the fetal liver. Long-term repopulating hematopoietic stem cells appear in the fetal liver around E12 [49]. Hematopoietic stem cells at this stage are highly proliferative. The cells double daily until E14 [49]. Notably, fetal liver hematopoietic stem cells preferentially differentiate into erythroid cells compared with adult bone marrow hematopoietic stem cells [50,51]. Fetal liver microenvironments partly explain erythroid bias because abundant erythropoietin seems to be expressed at this stage [52].

**Figure 3:**
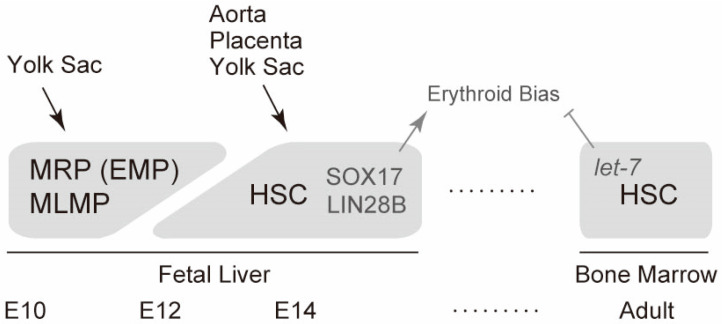
Cellular sources of definitive erythropoiesis. Timeline of definitive erythropoiesis during mouse development is schematically shown together with cellular sources of erythrocytes in each site of hematopoiesis. The molecules involved in erythroid bias are also indicated. MRP, myeloid-restricted progenitors; EMP, erythroid-myeloid progenitors; MLMP, multipotent lympho-myeloid progenitors; HSC, hematopoietic stem cells.

Recent studies have revealed that two molecules in fetal liver hematopoietic cells are involved in erythroid bias. The transcription factor SOX17 is one molecule that can explain erythroid bias. SOX17 is expressed in the fetal liver, but not in the bone marrow or hematopoietic stem cells [50]. Ectopic expression of SOX17 confers erythroid bias and a more-proliferative (self-renewable) phenotype in bone marrow hematopoietic stem cells [50]. The other molecule that accounts for erythroid bias is LIN28B. The heterochronic regulator LIN28 is an RNA-binding protein that post-transcriptionally inhibits the biogenesis of *let-7* microRNA [53]. Two paralogues of LIN28A and LIN28B are known. A subclass of *let-7* microRNA that has two separate binding motifs for a LIN28 protein is strongly inhibited [53]. LIN28B is highly expressed in the fetal liver compared with that in adult bone marrow hematopoietic progenitors, whereas *let-7* microRNAs are abundantly expressed in adult bone marrow hematopoietic cells [54]. Enforced expression of LIN28B or inhibition of *let-7* confers erythroid bias in bone marrow hematopoietic cells. Conversely, LIN28B deficiency or *let-7* transduction eliminates the erythroid bias of fetal liver hematopoietic cells [54]. A recent report showed that another RNA-binding protein, IGF2BP3, may take part in LIN28B-mediated regulation [55]. In addition, the epigenetic regulator EZH2 may act together with let-7 to repress fetal gene expression in the adult hematopoietic system [56]. Thus, mechanisms controlled by SOX17 and LIN28B distinguish fetal liver from adult bone marrow hematopoietic stem cells. A summarized schema for fetal liver erythropoiesis is illustrated in Figure 3.

## 6. Embryonic and Fetal Erythropoiesis in Humans

In humans, the initial formation of blood islands with erythroid cells is observed in the yolk sac of embryos aged 17 days [57]. Human primitive erythrocytes emerge as large-nucleated cells and express ζ and ε globins, as mentioned earlier. Human primitive erythrocytes enter into circulation around day 22 of embryonic age when the heart starts to beat [57]. Human hematopoietic stem cells that can be engrafted on mice for a long period are first observed around week 6 of human embryonic development [58,59]. Apparently, a large number of hematopoietic progenitors (in vitro colony forming cells) are present in the yolk sac and other portions of human embryos compared with that in transplantable hematopoietic stem cells [59]. These cells may partly represent cell types intermediate between primitive erythropoiesis and hematopoietic stem-cell-originated definitive hematopoiesis, similar to that in the mouse embryonic hematopoietic system mentioned earlier.

After the first trimester of gestation, primitive erythrocytes are replaced by fetal erythropoiesis in the fetal liver. Fetal erythrocytes express α- and γ-globins. Since γ-globins continue to be expressed throughout fetal life and are silenced after birth, hematopoietic stem cells in the fetal liver are considered to produce these fetal erythrocytes expressing α- and γ-globins [5]. It remains unknown whether “transient definitive” hematopoietic progenitors such as those in a mouse system contribute to the second wave of erythropoiesis in the human fetus.

Thus, the second globin switch (γ to β) is considered to occur in hematopoietic stem cell-derived erythroid cells. This switch within β globin genes is critical for clinical outcomes. β-Thalassemia is a disorder caused by reduced production or absence of β-globin whereby patients present anemia. β-Thalassemia patients with relatively high expression of γ fetal globin undergo a milder course of the disease [60]. Natural regulatory mutations that upregulate γ globin genes are located in the promoter region of the genes. Notably, the mutated sites in the γ globin genes are consistent with the BCL11A and ZBTB7A binding sites [12]. Therefore, modification of the binding of these transcription factors is a potential target for new therapies aimed at reactivating γ fetal globin genes in β-thalassemia patients [60,61]. A recent study reported that the above-mentioned LIN28B pathway regulates BCL11A translation in humans [62]. Furthermore, ATF4 and MYB are reported to upregulate the BCL11A gene [13]. Controlling these factors may also promote the reactivation of γ fetal globin genes.

The cell lineage tree of committed hematopoietic cells within the embryonic and fetal stages of human development is largely unknown. However, studies using in vitro cell cultures of human embryonic stem cells indicate that hemogenic endothelial cells are the initial source of primitive erythrocytes and multilineage blood cell types [63,64]. Future studies using human pluripotent stem cells may further reveal the details of the cellular pathways leading to the generation of primitive and definitive erythrocytes in human embryos.

## 7. Conclusions

Embryonic and fetal hematopoiesis do not occur in simple two-step phases of primitive erythropoiesis and definitive hematopoiesis. Rather, these are multiple developmental stages before hematopoietic stem cells start to constantly produce mature progenies in the fetal liver. Primitive erythrocytes are the first hematopoietic cells produced solely in the yolk sac during development. However, primitive erythrocytes are not completely discrete from lympho-myeloid lineages. Primitive erythroid cells and transient lympho-myeloid cells share a common developmental step during the embryonic stage. Multipotent hematopoietic progenitors originating in the yolk sac contribute to the initial and transient wave of definitive erythropoiesis and hematopoiesis in the fetal liver. Finally, hematopoietic stem cells that expand in the fetal liver take over these functions and become the predominant source of erythrocytes and other hematopoietic cells. Thus, embryonic and fetal hematopoiesis occur in a multi-step manner.

Recent studies have greatly advanced the understanding of hematopoiesis in the yolk sac. In particular, recent lineage tracking technologies have uncovered many new findings about macrophage lineages originating from the yolk sac [65,66,67,68]. However, it is not precisely known how a variety of hematopoietic cells in the yolk sac contribute to embryonic and fetal hematopoietic systems. Although recent trends using reporter systems have revealed new aspects of embryonic and fetal hematopoiesis, it seems that some more caution needs to be paid because some studies lack full characterization of the transgenic reporter system to determine whether the reporter faithfully recapitulates the original gene expression and where it is expressed. Further studies need to be performed to precisely describe embryonic and fetal hematopoiesis. Understanding the process of embryonic and fetal erythropoiesis may further bring new concepts into the phylogeny, ontogeny, and stem cell system of animal bodies.
